# Survival from cancers of the colon and rectum in England and Wales up to 2001

**DOI:** 10.1038/sj.bjc.6604580

**Published:** 2008-09-23

**Authors:** A G Acheson, J H Scholefield

**Affiliations:** 1 Section of Surgery, University of Nottingham, University Hospital, Floor E, West Block, Nottingham NG7 2UH, UK

Colorectal cancer is the third most common malignancy in the ‘developed’ world, after lung and breast cancer, and causes approximately 16 000 deaths per year in England and Wales. It usually occurs in the seventh or eight decade and has a similar overall incidence in the sexes. Despite improvements in early diagnosis, over recent years up to 30% of large bowel cancers still present as emergencies with obstruction or perforation and approximately 30% present late with metastatic disease.

Despite significant advancements in diagnostics, surgical technique, perioperative and postoperative therapies, many sceptics still suggest that the mortality from this disease has changed very little over the last few decades. The two preceding articles in this supplement of the *British Journal of Cancer* identify considerable improvements with regard to our management of patients with colorectal cancer in the United Kingdom. These papers report an increasing incidence rate of the disease but more important is the improvement in survival figures for colon cancer in England and Wales with a 40% 5-year survival rate in the late 1980s compared with 48% in the late 1990s. The improvement for rectal cancer is even more marked than for colon cancer. Although the absolute values of 5-year survival are inflated (as discussed by the authors) due to patients excluded from their analysis having a shorter survival period, the survival trends are most striking and encouraging.

So why has the incidence of colorectal cancer increased at the end of the 20th century? This is difficult to fully explain but there are a number of factors that may have contributed. Colonoscopic screening of high-risk individuals with a strong predisposition for colorectal cancer such as a strong family history, adenomatous polyps and inflammatory bowel disease has undoubtedly improved the detection rate of the disease (Atkin *et al*, 2002). Increased public awareness of the disease along with more willingness to report symptoms to the family doctor may be a significant factor, with consequent earlier presentation of cases. Dietary factors may be important with those consuming high meat, low-fibre diets felt to be at greater risk. Other lifestyle factors such as obesity, falling rates of physical activity and increasing consumption of alcohol may also have played a part.

And why have survival rates improved towards the latter part of the 20th century? Again increased public awareness leading to earlier diagnosis of better stage tumours is important. The introduction of the multidisciplinary team approach to the management of all patients with colorectal cancer has been instrumental in improving the decision making and eventual outcome of these patients (Kelly *et al*, 2003). Improved training workshops and specialisation of general surgeons has occurred with advances in surgical techniques, particularly in relation to rectal cancer, and this has resulted in the adoption of meticulous surgery in the form of total mesorectal excision (TME) with lower local recurrence rates and improved survival (MacFarlane *et al*, 1993). Similarly, advances in more effective adjuvant chemo- and radiotherapy have had a significant part to play, although the availability of many of the expensive adjuvant chemotherapeutic agents remains a subject of debate in the United Kingdom (Cammà *et al*, 2000; Tebbutt *et al*, 2002). Over the past 20 years there has been much enthusiasm in support of liver resection in patients with metastatic colorectal cancer with many centres reporting excellent results. Regular imaging and detection of operable liver lesions during staging and follow-up of colorectal cancer has also resulted in some survival benefit (Garden *et al*, 2006).

It is disappointing that survival rates among the more affluent remain better than among the more deprived. We agree with the authors that this suggests that more affluent patients have probably benefited preferentially from progress in early diagnostic procedures and in access to optimal treatment over this period, highlighting an important ongoing challenge for health services.

Despite the encouraging survival trends there is still room for improvement, particularly, as many of our American and European counterparts quote survival figures over 50% (O’Connell *et al*, 2004). The Department of Health has suggested that these UK statistics do not reflect any progress from the significant investment in cancer care since the publication of the NHS Cancer Plan in 2000. Government targets have been introduced in an attempt to identify ‘high risk or alarm symptoms’ with the aim of reducing delays between presentation, diagnosis and treatment. Time will tell if these target guidelines will bring any long-term survival benefits to patients in the United Kingdom.

Over the next few years bowel cancer screening by means of faecal occult blood (FOB) testing is being phased in throughout England and Wales. It is hoped that by implementing such a national population screening programme, many earlier stage cancers will be detected and this should reduce disease specific mortality as has been shown in several previous randomised controlled trials (Mandel *et al*, 1993; Hardcastle *et al*, 1996; Kronborg *et al*, 1996).

Despite the disappointing projected trends in survival from rectal cancer, important new developments in the management of the disease allow us to be more optimistic about continuing improved survival rates for colorectal cancer into the 21st century. The outstanding challenge for health services will be to ensure that the projected widening of the deprivation gap does not become a reality and to develop strategies to close this gap in forthcoming years.
